# Machine Learning-Based Prediction of Early Left Ventricular Function After STEMI

**DOI:** 10.3390/jcm14238563

**Published:** 2025-12-03

**Authors:** Shunjie-Fabian Zheng, Kathrin Diegruber, David Esser, Solveig Vieluf, Christopher Stremmel

**Affiliations:** 1 Department of Medicine I, LMU University Hospital, LMU Munich, 81377 Munich, Germanysolveig.vieluf@med.uni-muenchen.de (S.V.); 2 DZHK (German Centre for Cardiovascular Research), Partner Site Munich Heart Alliance, LMU University Hospital, LMU Munich, 81377 Munich, Germany

**Keywords:** ST-segment elevation myocardial infarction, LV function, lactate, risk prediction, artificial intelligence, machine learning

## Abstract

**Background:** Left ventricular (LV) function and lactate dynamics are major prognostic markers after ST-segment elevation myocardial infarction (STEMI). Early identification of patients at risk for impaired LV function or systemic hypoperfusion may improve outcomes. Machine learning (ML) can enhance predictive accuracy beyond traditional statistical methods, yet most prior studies were limited by small sample sizes and categorical outcomes. **Methods:** We retrospectively analyzed 2132 consecutive STEMI patients admitted to LMU Hospital (2014–2023). After preprocessing, 1608 patients with complete data were included. Thirty-eight demographic, clinical, procedural, and laboratory variables were used to train Decision Tree, Random Forest, and XGBoost regression models for predicting continuous left ventricular ejection fraction (LVEF) at discharge and lactate levels during hospitalization. Model performance was evaluated using mean squared error (MSE), root mean squared error (RMSE), mean absolute error (MAE), coefficient of determination (R^2^), and mean absolute percentage error (MAPE). Feature importance and Shapley additive explanations (SHAP) were applied for interpretability. **Results:** Ensemble models outperformed single trees. XGBoost achieved the best performance for LVEF prediction (MSE = 0.008, RMSE = 0.086, MAE = 0.068, R^2^ = 0.35). Lactate prediction showed moderate accuracy (R^2^ = 0.42 for admission and 0.47 for peak levels). Key predictors included cardiogenic shock, left anterior descending (LAD) culprit lesions, and peak lactate. **Conclusions:** ML enables individualized prediction of LV function and lactate dynamics after STEMI using routinely available clinical and laboratory data. Ensemble models, particularly XGBoost, demonstrated consistent and clinically meaningful predictive performance and generalizability, supporting their potential for early, data-driven risk stratification in acute cardiac care.

## 1. Introduction

Cardiovascular diseases remain the leading cause of morbidity and mortality worldwide, with acute myocardial infarction (MI)—particularly ST-elevation myocardial infarction (STEMI)—representing one of the most severe and life-threatening clinical manifestations [1]. Despite significant advances in reperfusion strategies and guideline-directed pharmacological therapy, STEMI continues to be associated with substantial short- and long-term complications, including heart failure, arrhythmias, and sudden cardiac death [2,3].

A central determinant of both early clinical course and long-term prognosis after STEMI is the left ventricular ejection fraction (LVEF). Reduced LVEF is a direct consequence of acute myocardial injury and remains one of the strongest predictors of morbidity, mortality, and arrhythmic events. It not only reflects the extent of ischemic damage but also guides treatment decisions, including the need for long-term heart failure therapy and prophylactic implantation of an implantable cardioverter-defibrillator (ICD), as outlined in international heart failure and post-MI management guidelines [2,3]. A machine learning (ML) study on one-year mortality risk prediction after STEMI identified LVEF as the strongest predictor of outcome (mean Shapley value (SHAP) of 0.978) in a cohort of 2887 patients [4].

Importantly, many patients who will eventually present with persistently reduced LV function show early signs already during the acute phase of STEMI—within the first 24 to 72 h. Hence, accurate early prediction of LVEF is clinically essential: it allows risk-adapted decisions regarding monitoring intensity (e.g., telemetry, intensive care unit (ICU) stay), timing of imaging studies, and early initiation of prognostically relevant treatments. Furthermore, in the face of growing pressure on healthcare resources, timely risk stratification also supports more efficient use of ICU capacity and helps avoid unnecessary prolongation of hospitalization.

Numerous clinical and biochemical markers have been investigated as predictors of post-infarct LVEF. These include patient-related factors (such as age, comorbidities, and Killip class), infarct characteristics (location, extent, and time to reperfusion), and laboratory values (e.g., creatine kinase (CK), CK-myocardial band (MB), and cardiac troponins) [5,6,7,8]. Several studies have shown that prolonged ischemia time and elevated peak biomarker levels, especially troponin, strongly correlate with infarct size and are independently associated with reduced LVEF [5,7,9,10,11]. Hemodynamic compromise on admission—manifested as hypotension, tachycardia, or cardiogenic shock—also reflects greater myocardial involvement and carries a high risk of LV dysfunction and adverse events [6].

Logistic regression models combining multiple parameters to predict LVEF showed promising results in previous analyses, but were limited by sample size and the number of assessed parameters [5,12,13]. Felbel and colleagues identified anterior wall myocardial infarction and pain-to-balloon time as the most decisive factors [5]. With respect to laboratory markers Reinstadler et al. predicted LV remodeling with an area under the curve (AUC) of 0.85 by a combination of lactate dehydrogenase (LDH), troponin, aspartate transaminase (AST), alanine transaminase (ALT), and N-terminal pro-B-type natriuretic peptide (NT-proBNP) measurements. Among these parameters troponin (AUC = 0.75) and LDH (AUC = 0.78) achieved the best individual results [13].

In contrast, while imaging-based predictors—such as wall motion abnormalities on echocardiography, strain analysis, or infarct size and microvascular obstruction on cardiac magnetic resonance imaging (CMR)—are known to be valuable, they require advanced diagnostics that are not always immediately available in the acute phase [14,15]. Likewise, advanced electrocardiographic parameters (e.g., ST-segment resolution, QRS fragmentation) and laboratory markers of wall stress (NT-proBNP) have shown prognostic value in selected cohorts, but were neither systematically assessed nor uniformly available in the present dataset [16,17,18]. Hence, these variables were excluded from the analysis in the current study. Similarly, we excluded inflammatory markers (e.g., leukocyte count, C-reactive protein (CRP)) due to their limited predictive value in relation to LV function and cardiovascular risk [19,20,21].

Despite the recognized importance of early LV function, there is still a lack of robust and clinically applicable prediction tools based solely on readily available parameters in the acute care setting. In recent years, artificial intelligence (AI) and ML approaches have shown great potential in the field of cardiovascular risk prediction. These methods can incorporate large numbers of variables and uncover complex, non-linear associations that may be missed by traditional statistical models. Initial studies applying ML to post-MI prognosis have reported promising predictive accuracy, suggesting that AI may augment clinical decision-making in this context. However, one of the largest studies on this topic included 1220 patients with myocardial infarction and focused on long-term prognosis. It achieved remarkable predictive power with the Extreme Gradient Boosting (XGBoost) model (AUC = 0.92) in relation to the development of heart failure. The leading predictive parameters based on SHAP analyses were LV function, LV end-systolic diameter, and LDH [22].

Short-term studies to optimize care capacities and to identify high-risk groups based on ML models are very limited. Jeon and colleagues used an AI-enabled ECG index and achieved promising results in predicting LV function with a cohort of 637 STEMI patients [23]. Similarly, a study by Xin and colleagues investigated 56 variables of 315 STEMI patients to predict infarction size. The Random Forest model achieved a coefficient of determination (R^2^) of 0.687 and the following ten factors were the most predictive parameters in feature importance analysis: leukocyte count, anterior wall MI, CK, cholesterol, troponin, platelet count, systolic blood pressure, blood urea nitrogen (BUN), creatinine, and NT-proBNP on admission [24]. Of note, LDH—a major predictive parameter in previous trials—was not included in this analysis and the overall sample size was relatively small [13,24].

The largest present study on short-term risk evaluation included 1863 patients to predict in-hospital mortality (AUC = 0.79), ICU admission (AUC = 0.78), and LVEF < 40% (AUC = 0.74). However, the predictive capacity of the model was limited due a limited number of evaluated parameters, namely age, pre-hospital cardiac arrest, robust collateral recruitment, cardiovascular risk factors, blood pressure, heart rate, culprit lesion, and TIMI flow [25]. Although laboratory values are known as the best predictors according to the current literature, they were not included in this trial.

Notably, most previous ML approaches have framed post-STEMI LVEF as a categorical outcome (e.g., reduced vs. preserved function), rather than as a continuous variable. While classification facilitates risk grouping, it oversimplifies the spectrum of ventricular dysfunction and may obscure subtle prognostic differences. Continuous prediction models, in contrast, allow a more precise and individualized estimation of LV function, enhancing clinical interpretability and risk stratification.

Our present study aims to systematically identify early predictors of LV function following STEMI by analyzing one of the largest retrospective STEMI cohorts to date. We applied ML algorithms, including multivariate regression models, to determine which combinations of clinical, laboratory, and procedural variables best predict continuous LVEF at hospital discharge. While previous studies have demonstrated the prognostic importance of LVEF and explored ML-based prediction approaches, existing models often relied on small samples, categorical outcomes, or advanced imaging modalities that limit clinical applicability.

In this study, we sought to overcome these limitations by developing and validating ML models for the continuous prediction of LVEF and lactate dynamics after STEMI using only routinely available clinical and laboratory data. We hypothesized that ensemble models based on such parameters could accurately estimate early LV function and identify patients at risk. Beyond methodological development, this study aimed to evaluate the feasibility of integrating these ML-based tools into clinical workflows to enable early, data-driven risk stratification in acute cardiac care.

## 2. Materials and Methods

### 2.1. Study Population

**Figure 1:**
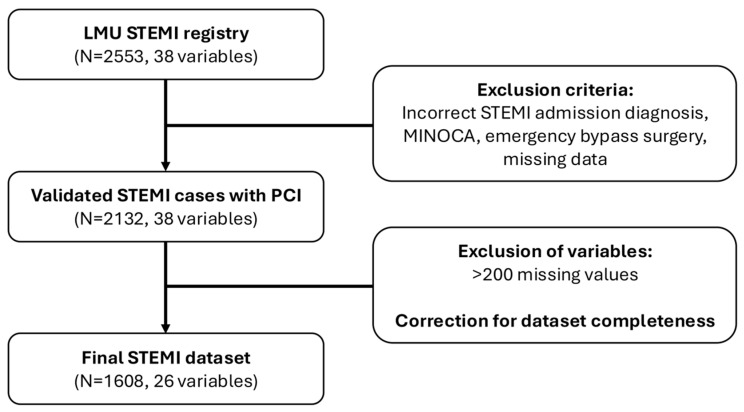
Patient and data selection flow chart. Depicted are patient cohorts and the number of variables on all selection levels. Major exclusion criteria for each step are highlighted in the boxes on the right. LMU, Ludwig-Maximilians-University; STEMI, ST-segment elevation myocardial infarction; PCI, percutaneous coronary intervention.

This retrospective cohort study was conducted at Ludwig-Maximilians-University (LMU) Hospital, Munich, Germany. We screened 2553 consecutive patients admitted with a primary diagnosis of STEMI between 2014 and 2023. Exclusion criteria included incorrect STEMI admission diagnosis, myocardial infarction with non-obstructive coronary arteries (MINOCA), indication for emergency bypass surgery, and incomplete datasets. After applying these criteria, 2132 patients remained eligible for our analysis. To ensure a fair comparison among different ensemble methods, we also drop predictors with more than 200 missing values (Figure 1, Supplementary Table S1).

This study was carried out in accordance with the Declaration of Helsinki and the German Data Protection Act. It was approved by the institutional ethics committee of LMU Munich (#23-0609).

### 2.2. Data Collection

The final STEMI registry comprised 2132 patients with a total of 38 variables. Specifically, features comprised demographics, cardiovascular risk factors, clinical history and course, procedural data, and laboratory markers. Variables with >200 missing values were excluded in a preprocessing step, resulting in a total of 26 analyzed variables, coded into 42 covariates (33 when excluding baseline category) considering the categorical nature of some variables (Supplementary Figure S1, Supplementary Table S1).

### 2.3. Echocardiographic Assessment of Left Ventricular Function

LVEF at discharge was assessed using transthoracic echocardiography performed in accordance with current guideline recommendations [26,27]. Standard apical two- and four-chamber views were acquired, and LVEF was calculated using the biplane Simpson’s method whenever image quality permitted. All examinations were conducted by board-certified cardiologists or experienced sonographers as part of routine clinical care. LVEF values used for the analysis were extracted from the finalized echocardiography reports stored in the institutional imaging archive. Inter-observer variability is expected to be minimal due to standardized acquisition and reporting protocols applied within our department.

### 2.4. Data Processing

Continuous variables were log-transformed (log + 1) to reduce skewness, to stabilize variance, and for normalization. Categorical variables were one-hot encoded to ensure comparability across models. Although some algorithms can process categorical features directly, consistent preprocessing was chosen to standardize analyses across methods (Supplementary Table S1).

It is important to note that we did not perform feature elimination. Because the ensemble methods used in this study do not rely on matrix inversion for coefficient estimation, multicollinearity does not pose a methodological limitation. Tree-based models are inherently robust to correlated predictors, and their predictive performance is not adversely affected by multicollinearity. In addition, one-hot encoding introduces correlations among dummy variables by design. Arbitrarily removing such variables through recursive elimination would be counterproductive, as it may exclude clinically relevant information without offering methodological benefit. To preserve the full clinical context and ensure that the models had access to the complete set of available predictors, all variables were retained [28,29] (Supplementary Figure S2).

### 2.5. Outcomes

The primary outcome was LV function at hospital discharge, a clinically relevant predictor of cardiovascular risk. LV function was directly taken as a continuous outcome.

Secondary outcomes were lactate concentrations, analyzed both at admission and at peak levels during hospitalization. Lactate was modeled analogously to LV function and (log + 1) transformed to tackle large outliers.

### 2.6. Model Development

For the complete dataset, predictors and biomarkers with >200 missing entries were excluded. Further, patients with missing values in the remaining covariates were excluded, yielding 1608 patients for model development. Data were randomly split into training (80%) and test (20%) sets (seed = 42). Missing data handling and preprocessing were performed before splitting. Hyperparameters were optimized using grid search with leave-one-out cross-validation, (n-1)-fold, on the test set [30].

We implemented regression models using Decision Trees, Random Forest, and XGBoost. To provide meaningful baseline benchmarks and contextualize the performance of the ensemble models, we additionally incorporated three linear models: Ordinary Least Squares Regression, Lasso Regularization (L1), and Ridge Regularization (L2). These linear baselines allow for a clearer comparison between traditional linear approaches and the non-linear ensemble methods applied in this study.

- Decision Trees: partition the feature space recursively to minimize variance within subgroups [31].
- Random Forests: ensemble of Decision Trees built on bootstrapped samples with feature subsampling to reduce variance and overfitting [29].
- XGBoost: sequentially adds trees to correct residual errors, with gradient-based optimization and regularization to control model complexity [28].

### 2.7. Model Evaluation

Regression performance was assessed using mean squared error (MSE), root mean squared error (RMSE), mean absolute error (MAE), coefficient of determination (R^2^), explained variance score (EVS), and mean absolute percentage error (MAPE). The model was validated on a temporally distinct dataset from the same institution (20 consecutive STEMI patients in January 2024).

### 2.8. Explainability

To ensure interpretability, we computed model-based feature importance scores and analyzed SHAP (Shapley additive explanations) values for global and local interpretability. Beeswarm plots were generated to visualize feature effects, illustrating both magnitude and direction of influence on a single patient and full cohort level.

### 2.9. Statistical Analysis

In a quest of analyzing differences across the patients with prior PCI and those without prior PCI, we deployed statistical tests. Continuous variables were summarized as mean (±SD). Binary variables were reported as proportions. Comparisons between groups (e.g., patients with vs. without prior intervention) were performed using the Mann–Whitney U test for continuous data and the χ^2^ or z-test for categorical variables.

### 2.10. Software

All analyses were performed using Python 3 (Python Software Foundation). Data preprocessing and management were carried out with the Pandas library (version 1.5) and NumPy (version 1.23) for numerical operations [32,33].

Machine learning models were implemented using the following:

- Scikit-learn (version 1.2) for Decision Tree and Random Forest regressors [34].
- XGBoost (version 1.7) for gradient boosting regression [28].

Model evaluation metrics, including MSE, RMSE, MAE, R^2^, EVS, and MAPE, were calculated using scikit-learn.

Visualization of results was performed using Matplotlib library (version 3.6) [35]. For model interpretability, we employed the SHAP library (version 0.41) to generate SHAP values, including beeswarm and summary plots [36].

Statistical analyses were conducted with scipy.stats (version 1.9) for nonparametric tests (Mann–Whitney U) and statsmodels (version 0.13) for parametric testing (proportional z-test) [37,38].

## 3. Results

### 3.1. Data Basis

Our study included a total of 2553 consecutive STEMI patients admitted to our hospital between 2014 and 2023. After exclusion of patients with an incorrect STEMI admission diagnosis, cases without acute coronary obstruction (MINOCA cohort), and patients with an indication for emergency bypass surgery, as well as missing datasets, 2132 patients with 38 different variables remained for further statistical evaluations.

Among these, variables with more than 200 missing values were excluded, resulting in 26 remaining variables. Further, we removed patients with missing values for a fair comparison, since XGBoost can handle missing values internally but other ensemble methods do not. This leads to a final complete dataset for 1608 patients (Figure 1, Supplementary Table S1, Supplementary Figure S1).

### 3.2. Baseline Characteristics

Our final study population included 1608 individuals with an average age of 65 years. Mean body mass index was 27.2 kg/m^2^ and one quarter of them were female (25.1%). About 20% (N = 325/1608) had a known coronary artery disease with a history of a prior percutaneous coronary intervention (PCI) (Table 1).

For the overall cohort, approximately 30% have coronary artery disease (CAD) affecting one (30.9%) or two vessels (27.8%), respectively, while the remaining 40% presented with a three-vessel pathology (41.3%). However, in patients with a prior PCI the relative proportion of three-vessel CAD was almost twice as high as in those without a prior PCI (64.0% vs. 35.5%; *p* < 0.001) (Table 1).

The culprit lesion was the left anterior descending artery (LAD) in half of all cases, right coronary artery (RCA) in one third, and left circumflex artery (LCX) in about 11%. The left main coronary artery required only very few PCIs in our cohort, with about 1–2%. Interestingly, the culprit lesion distribution was independent of the status regarding a prior PCI (Table 1).

Procedural data indicated an average contrast agent volume (CAV) of about 207 ± 98 mL with a radiation time of 14.1 ± 11.7 min and a dose area product (DAP) of 4306 ± 4111 cGy/cm^2^. Tirofiban, a potent intravenous antiplatelet agent, was used in 10% of cases due to very high thrombus load or lack of reperfusion. Of note is that the use of tirofiban was more frequent in patients with prior PCI (16.6% vs. 8.3%; *p* < 0.001) (Table 1).

### 3.3. Hemodynamics and Shock

A total of 16% of all patients presented with cardiogenic shock on admission with a significantly higher proportion among those with prior PCI (23% vs. 15%; *p* = 0.001). Cardiopulmonary resuscitation (CPR) was necessary in 14% and in about 5% mechanical circulatory support (MCS) systems were implanted. These were clearly dominated by extracorporeal membrane oxygenation (ECMO) systems (4.7% of patients) and a minor proportion of Impella (0.9%), ECMO + Impella (0.3%), or intra-aortic balloon pump (IABP) devices (0.3%). Importantly, the LVEF upon discharge was only mildly impaired, at 49 ± 11% (Table 1).

### 3.4. Laboratory Indicators of Infarction Size

Key laboratory findings indicate high CK values on admission (875 ± 1502 U/L) with a maximum peak of 2348 ± 3727 U/L reflecting a significant myocardial infarction. This goes in line with cardiac-specific troponin T values of 3.4 ng/mL on admission, which rose up to 9.1 ng/mL in the further clinical course. LDH as a marker of general tissue damage was 421 ± 653 U/L on admission and average renal function was largely normal (creatinine 1.13 ± 0.62 mg/dL) in our cohort. In line with only a minor proportion of STEMI patients presenting with cardiogenic shock, lactate values were only mildly elevated in our cohort on admission (2.48 ± 2.47 mmol/L), as were their maximum peaks (3.49 ± 3.80 mmol/L).

When stratified for the presence of a prior PCI, we identified slightly higher infarction sizes based on laboratory markers in patients with no prior PCI (CK max 2361 vs. 2296 U/L, *p* = 0.013; CK-MB max 241 vs. 207 U/L, *p* = 0.001), although maximum troponin T as the best investigated marker did not reach statistical significance (troponin T max 9.04 vs. 9.25 ng/mL; *p* = 0.059). Moreover, renal function based on creatinine measurements was slightly worse in patients with a prior PCI.

### 3.5. Mortality

Overall mortality for our final STEMI cohort was 6.6% (N = 106/1608). In line with previous studies, we set the LVEF threshold to 40% and divided our study cohort into two groups [25]. While STEMI patients with an LVEF of 40% had a mortality rate of 2.6% (N = 36/1374), it was 29.9% (N = 70/234) in patients with an LVEF < 40% (*p* < 0.001).

### 3.6. Model Performance in Prediction of LV Function

For the prediction of LVEF based on our dataset, the Decision Tree model showed the weakest performance, with an MAE of 0.075, and an R^2^ of only 0.17 (MSE = 0.010, RMSE = 0.097). In contrast, the ensemble approaches yielded markedly better results. Random Forest achieved an MAE of 0.067 and an R^2^ of 0.33 (MSE = 0.008, RMSE = 0.087). XGBoost provided the most favorable overall performance, with an MAE of 0.068 and an R^2^ of 0.35 (MSE = 0.008, RMSE = 0.086). Although the differences between Random Forest and XGBoost were small, both methods substantially outperformed the single Decision Tree, highlighting the advantage of ensemble learning for this prediction task.

Furthermore, the linear baseline models—Ordinary Least Squares Regression, Lasso Regularization (L1), and Ridge Regularization (L2)—demonstrated competitive error metrics but consistently explained less variance than the ensemble methods. This performance gap highlights the limitations of traditional linear approaches in modeling non-linear relationships within the dataset and further emphasizes the benefit of ensemble-based techniques for LVEF prediction (Table 2).

In the stratified analysis according to prior PCI, model performance differed substantially between subgroups. In patients with previous PCI, all models showed lower predictive accuracy, with XGBoost achieving the highest R^2^ of 0.24 but with markedly higher error metrics (RMSE = 0.107, MAPE = 22.9%) compared to the overall cohort. In contrast, in patients without prior PCI, predictive performance improved, with both Random Forest (R^2^ = 0.36, RMSE = 0.085, MAPE = 15.6%) and XGBoost (R^2^ = 0.35, RMSE = 0.086, MAPE = 15.8%) achieving substantially better results. These findings suggest that the presence of prior PCI may be associated with increased heterogeneity in LV function, rendering accurate prediction from clinical variables more challenging (Table 2).

**Figure 2:**
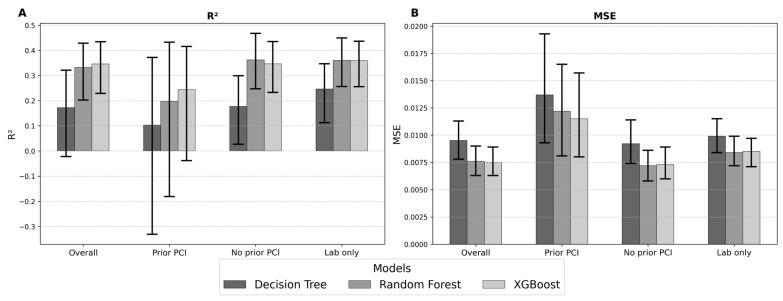
Model performance for LVEF prediction. Comparison of Decision Tree, Random Forest, and XGBoost models for the prediction of the exact LVEF function for the full cohort of patients as well as in relation to the presence or absence of a prior percutaneous coronary intervention (PCI) or based on laboratory values only. The models are evaluated by (**A**) the R^2^ (the higher the better) and (**B**) the MSE (the lower the better). The performance is shown with the confidence intervals in red.

When restricting the predictor set to laboratory parameters only, model performance was comparable, though with some notable differences, to that obtained from the full clinical dataset. The Decision Tree achieved limited accuracy (MSE = 0.010, RMSE = 0.100, MAE = 0.080, R^2^ = 0.25, MAPE = 19.7%), whereas both Random Forest (MSE = 0.008, RMSE = 0.092, MAE = 0.074, R^2^ = 0.36, MAPE = 18.0%) and XGBoost (MSE = 0.009, RMSE = 0.092, MAE = 0.074, R^2^ = 0.36, MAPE = 18.2%) demonstrated superior predictive performance. Interestingly, R^2^ in the laboratory-only setting was slightly higher than in the overall cohort, suggesting that laboratory markers capture a substantial proportion of the variability in LV function. However, error metrics such as RMSE and MAE were consistently higher, indicating lower precision of individual predictions when non-laboratory clinical variables were excluded. Taken together, these results highlight that while laboratory markers alone provide valuable predictive information, integration with broader clinical data improves the accuracy of LV function estimation (Figure 2, Table 2).

### 3.7. Categorical Analysis of LV Function

To facilitate comparability with previous work, we additionally performed a categorical analysis of the LV function and applied separators at LVEFs of 30%, 35%, 40%, and 50%, respectively (Table 3, Supplementary Figure S3). Overall, an LVEF threshold of 40% provided the best results with an AUC of 0.82 (with logistic regression (LR); XGBoost AUC = 0.80) in our model—a threshold that has also proven to be optimal in the largest previous studies on this topic [25]. In line with our data on continuous LVEF, the model performs even better in patients without a prior PCI (XGBoost AUC = 0.89) and still provides good results when exclusively based on laboratory values (XGBoost AUC = 0.80) (Table 3, Supplementary Figure S3).

### 3.8. Lactate Values as a Surrogate for LV Function

LV function is a major determinant of cardiac output and overall hemodynamic stability. Impaired LV function may lead to tissue hypoperfusion, which is often reflected by elevated lactate concentrations. Given this close pathophysiological relationship, we investigated whether ML models trained on clinical and laboratory parameters could predict lactate levels both at hospital admission and at their peak values during the clinical course. This concept is in line with several studies reporting a strong correlation between lactate levels and cardiovascular outcome [39,40,41]. We know lactate prediction has no direct application in the clinical setting due to fast and easy point of care measurements. Yet we used this important parameter to evaluate the overall performance of our model.

**Figure 3:**
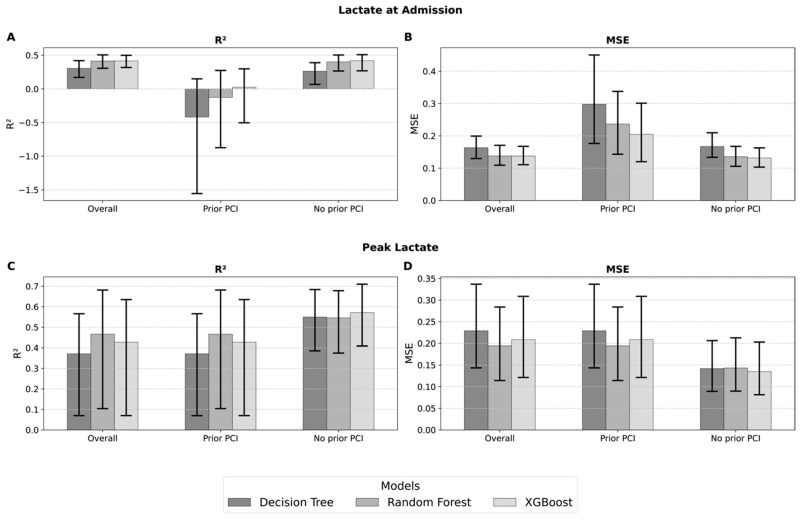
Model performance for lactate prediction. Comparison of Decision Tree, Random Forest, and XGBoost models for the prediction of log lactate values across all patients (overall) and in relation to the presence or absence of a prior percutaneous coronary intervention (PCI). The first row contains the prediction performance for the log admission lactate with (**A**) the R^2^ and (**B**) MSE. The second row shows the prediction performance of the log peak lactate with (**C**) the R^2^ and (**D**) MSE.

For the prediction of lactate levels at hospital admission, ensemble models outperformed a single Decision Tree. In the overall cohort, Random Forest (MSE = 0.138, RMSE = 0.372, MAE = 0.274, R^2^ = 0.41, EVS = 0.42, MAPE = 27.2%) and XGBoost (MSE = 0.137, RMSE = 0.371, MAE = 0.274, R^2^ = 0.42, EVS = 0.42, MAPE = 27.4%) demonstrated superior predictive accuracy compared to the Decision Tree (R^2^ = 0.30, MAPE = 29.4%). Subgroup analysis revealed considerable heterogeneity: In patients with prior PCI, prediction accuracy was low, with negative R^2^ values for Decision Tree and Random Forest and only marginal performance of XGBoost (R^2^ = 0.02, MAPE = 39.1%). In contrast, in patients without prior PCI, both Random Forest and XGBoost achieved consistent moderate predictive performance (R^2^ = 0.40 and 0.42, respectively; MAPE = 28%), comparable to or slightly better than the full cohort. These findings suggest that lactate levels on admission can be predicted with moderate accuracy, particularly in patients without prior PCI, whereas prediction is substantially less reliable in those with a history of PCI (Figure 3, Supplementary Table S2).

For the prediction of peak lactate values during the clinical course, ensemble models again outperformed a single Decision Tree. In the overall cohort, Random Forest yielded the best performance (MSE = 0.194, RMSE = 0.441, MAE = 0.333, R^2^ = 0.47, EVS = 0.51, MAPE = 29.6%), closely followed by XGBoost (R^2^ = 0.43, MAPE = 30.8%), whereas the Decision Tree performed worse (R^2^ = 0.37, MAPE = 28.9%). Subgroup analysis revealed that in patients without prior PCI, predictive performance improved substantially, with XGBoost achieving R^2^ = 0.57, RMSE = 0.368, and MAPE = 19.4%. By contrast, in patients with prior PCI, performance was reduced, mirroring the overall cohort results. These findings indicate that peak lactate levels can be predicted with moderate accuracy, particularly in patients without a history of PCI, while prediction remains less reliable in those with prior PCI (Figure 3, Supplementary Table S3).

A categorical analysis of admission lactate values with cutoffs of 2.5, 3–0, and 3.5 mmol/L provided the best values for the 3.5 mmol/L cutoff. The LR for the full cohort resulted in AUCs of 0.88 and 0.76 when analyzed with XGBoost. Like our LV function analysis, patients without a prior PCI allowed better prediction of admission lactate values (XGBoost AUC = 0.90). Results for peak lactate values were largely comparable to admission values for the full cohort (LR AUC = 0.84) as well as in relation to the presence or absence of a prior PCI (prior PCI Random Forest AUC = 0.93; no prior PCI Random Forest AUC = 0.84) (Supplementary Tables S4 and S5).

### 3.9. Feature Importance

**Figure 4:**
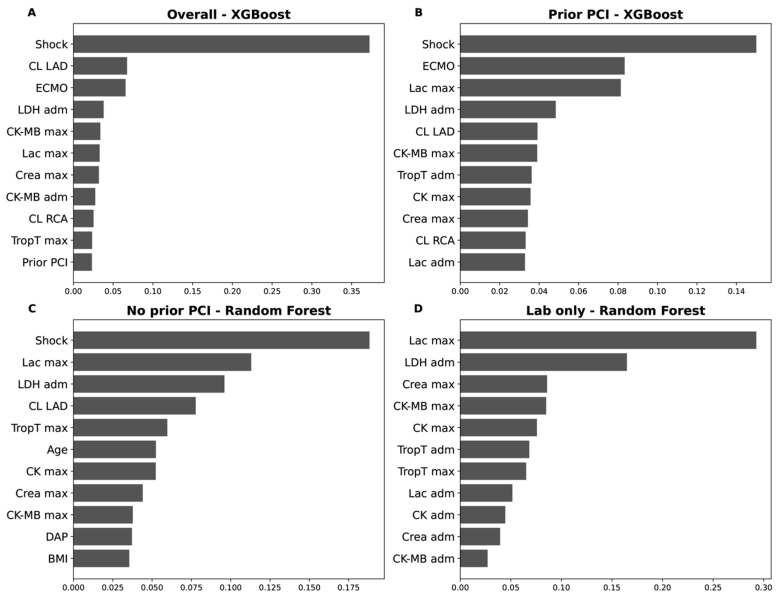
Feature importance for LVEF prediction. Depicted are variables with the highest feature importance values for (**A**) the overall cohort, in relation to (**B**) the presence or (**C**) absence of a prior percutaneous coronary intervention (PCI) and (**D**) based on laboratory values only. CL, culprit lesion; LAD, left anterior descending coronary artery; ECMO, extracorporeal membrane oxygenation; LDH, lactate dehydrogenase; adm, admission; CK, creatine kinase-myocardial band; MB, myocardial band; max, maximum; Lac, lactate; Crea, creatinine; RCA, right coronary artery; TropT, troponin T; DAP, dose area product; BMI, body mass index.

**Figure 5:**
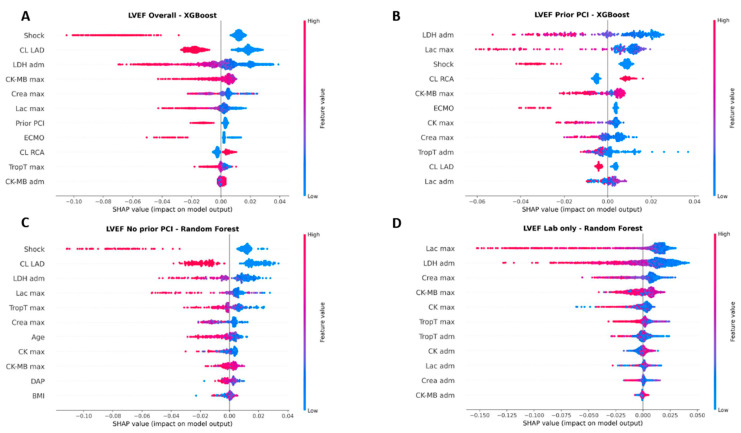
SHAP beeswarm of XGBoost regression for LVEF prediction. Depicted data were trained on (**A**) the full cohort as well as in relation to (**B**) the presence or (**C**) absence of a prior percutaneous coronary intervention (PCI) and (**D**) based on laboratory values only. CL, culprit lesion; LAD, left anterior descending; LDH, lactate dehydrogenase; adm, admission; max, maximum; CK, creatine kinase; MB, myocardial band, Crea, creatinine; Lac, lactate; ECMO, extracorporeal membrane oxygenation; RCA right coronary artery; TropT, troponin T; DAP, dose area product; BMI, body mass index.

The feature importance and SHAP beeswarm plots display the most influential covariates in the best performing models. We explicitly drop features of very low importance. In our analysis, the presence of cardiogenic shock emerged as the strongest predictor of LV function. This finding is not surprising, as cardiogenic shock in the STEMI population typically reflects an acute impairment of LV function that often does not fully recover even after successful revascularization of the culprit vessel. In some cases within our cohort, shock severity necessitated the implantation of a MCS device, which also explains the close association with ECMO implantation (Figure 4 and Figure 5).

Among the coronary vessels, a culprit lesion in the LAD had the largest impact on subsequent LV function, which is consistent with its central role in myocardial perfusion. When focusing on laboratory parameters, peak lactate levels showed high feature importance, which is in line with their established role as a surrogate marker of cardiogenic shock and systemic hypoperfusion [39,42,43]. Interestingly, LDH at admission also demonstrated strong predictive value. This may be explained by two factors: first, LDH serves as a nonspecific marker of tissue injury, and second, it may reflect the time delay between ischemia onset and hospital admission. Higher LDH levels therefore indicate longer ischemia duration, correlating with irreversible myocardial damage and subsequently reduced LV function (Figure 4 and Figure 5).

Classical cardiac biomarkers such as troponin T, CK, and CK-MB all exhibited predictive value without one parameter clearly outperforming the others. Notably, serum creatinine levels were also associated with later LV function. This association may reflect a higher overall morbidity in patients with renal impairment, but it could also be a consequence of true renal hypoperfusion secondary to reduced LV function (Figure 4 and Figure 5).

An analogous feature importance analysis including SHAP beeswarm with respect to lactate levels at admission and their peak values during the clinical course yielded very similar results. However, CPR emerged as an additional important variable in this context. This is most likely explained by the fact that prior CPR episodes can substantially elevate lactate levels, while having only a limited direct impact on subsequent LV function (Supplementary Figure S4).

### 3.10. Independent Validation

To further evaluate model robustness, we performed an additional validation in 20 consecutive STEMI patients admitted in January 2024, after register completion (Table 4). For the full cohort, XGBoost achieved comparable performance to the model development cohort (MSE = 0.0080, RMSE = 0.089, MAE = 0.079, R^2^ = 0.34, EVS = 0.37, MAPE = 18.5%), closely matching our overall results (R^2^ = 0.35, MAPE = 16.1%). In the subgroup of patients with prior PCI, predictive accuracy was substantially lower, with the validation yielding a negative explained variance (R^2^ = −0.56) despite moderate error metrics (RMSE = 0.099, MAPE = 22.6%), consistent with the lower performance already observed during model development (R^2^ = 0.24). In patients without prior PCI, Random Forest maintained good predictive accuracy (R^2^ = 0.29, MAPE = 18.6%) in subsequent validation, although slightly lower compared with model development results (R^2^ = 0.36, MAPE = 15.6%). Similarly, when restricting predictors to laboratory values only, Random Forest achieved moderate accuracy in validation (R^2^ = 0.25, MAPE = 20.2%), somewhat lower than in the model development setting (R^2^ = 0.36, MAPE = 18.0%) (Table 4).

For the prediction of lactate levels at admission, our validation cohort demonstrated consistent but heterogeneous results across subgroups. In the full cohort, XGBoost achieved an MSE of 0.0800, RMSE of 0.283, MAE of 0.239, R^2^ of 0.19, EVS of 0.27, and MAPE of 19.6%. While error metrics improved compared with the model development cohort (RMSE = 0.371, MAE = 0.274, MAPE = 27.4%), the explained variance was lower for the independent validation group (R^2^ = 0.42 in the model development cohort). In patients with prior PCI, predictive accuracy remained poor, with validation yielding a negative R^2^ (−0.15) and high error values (RMSE = 0.411, MAPE = 35.9%), consistent with the weak performance observed in the model development cohort (R^2^ = 0.02, MAPE = 39.1%). By contrast, in patients without prior PCI, validation demonstrated robust performance, with R^2^ = 0.43 and MAPE = 15.7%, closely mirroring or even improving upon the model development results (R^2^ = 0.42, MAPE = 27.6%) (Supplementary Table S6).

For the prediction of peak lactate levels, validation confirmed the overall trends observed during model development. In the full cohort, Random Forest achieved an MSE of 0.178, RMSE of 0.422, MAE of 0.312, R^2^ of 0.40, EVS of 0.41, and MAPE of 21.8%. This performance was comparable to the model development cohort (R^2^ = 0.47, MAPE = 29.6%), with slightly lower error metrics. In patients with prior PCI, however, predictive performance deteriorated markedly, with our validation cohort yielding an RMSE of 0.731, an MAE of 0.637, negative explained variance (R^2^ = −0.74, EVS = −0.62), and an MAPE of 46.3%, indicating poor model generalizability in this subgroup. By contrast, in patients without prior PCI, XGBoost maintained robust predictive accuracy, with validation yielding MSE = 0.116, RMSE = 0.341, MAE = 0.265, R^2^ = 0.60, EVS = 0.61, and MAPE = 17.7%, closely aligning with or slightly improving upon the results from model development (R^2^ = 0.57, MAPE = 19.4%) (Supplementary Table S7).

## 4. Discussion

This study demonstrates the feasibility of predicting LV function and lactate concentration after STEMI using routinely available clinical and laboratory parameters with ML approaches. In contrast to prior STEMI ML studies that typically enrolled a few hundred to about 1800 patients and often focused on classification rather than exact-value prediction, our single-center cohort (>2100 screened; 1608 for modeling) is among the largest to date for this purpose and enabled extensive subgroup analyses (prior PCI vs. no prior PCI; laboratory-only features) [20,21,22]. Beyond LV function, we extend prediction to admission and peak lactate, a marker closely linked to hemodynamic compromise and short-term prognosis [39,40,41,42,43].

Compared with earlier work emphasizing classical regression or restricted biomarker panels [5,12,13], we integrated a broad array of demographic, clinical, procedural, and laboratory variables and observed consistent gains of ensemble methods (Random Forest, XGBoost) over single trees [28,29,31], in line with method-agnostic benchmarks showing that tree-based models remain state-of-the-art for medium-sized tabular datasets [44,45].

In addition to these findings, recent advances in artificial intelligence highlight promising opportunities for future multimodal extensions of our approach. Several contemporary studies have demonstrated that combining ECG signals, echocardiographic imaging, and clinical data can substantially improve cardiac risk stratification and functional assessment. Parvathi et al. showed that integrating ECG images with clinical variables via a hybrid convolutional neural network-ensemble architecture improves myocardial infarction prediction, while Soto et al. demonstrated that a multimodal deep learning framework using 12-lead ECGs and echocardiogram videos enhances diagnostic precision for left ventricular hypertrophy [46,47]. Similarly, other investigations have applied deep learning algorithms to echocardiographic video sequences or 12-lead ECG recordings to quantify cardiac function, further illustrating the potential of imaging- and signal-based approaches [23,48,49]. Broader methodological reviews further emphasize that multimodal AI integrating imaging, physiologic signals, and structured clinical data is increasingly used across cardiovascular medicine [50,51].

Beyond imaging- and ECG-based prediction, artificial intelligence has also shown utility across other areas of cardiology. The recent perspective by Cersosimo et al. discusses emerging applications of AI and large language models in electrophysiology, including ECG interpretation, arrhythmia detection, ablation guidance, and sudden cardiac death risk stratification [52]. Incorporating these studies provides broader clinical context and situates our work within the rapidly expanding adoption of AI-driven tools in cardiovascular care. While our present models rely solely on routinely collected clinical and laboratory data, these multimodal approaches outline future directions for extending our methodology and potentially improving predictive performance even further.

Our focus on exact-value LVEF prediction complements prior classification-oriented models (e.g., LVEF < 40% or in-hospital endpoints) and AI-enabled ECG indices for LV dysfunction in STEMI, supporting the incremental value of routine, early-available signals [20,22]. We achieved an MAE of 7% for LVEF prediction—even if the model was exclusively based on laboratory values. Of note is that routine quick LVEF estimation in the emergency department or intensive care unit by visual approximation reaches the same accuracy of 7% if performed by experienced examiners [53,54,55]. Integrating such predictive models into clinical information systems or mobile decision-support dashboards could improve real-time patient triage.

In the benchmark analysis with previous ML studies our model demonstrates robust performance. In a classification-oriented model, i.e., predicting an LVEF < 40%, Sritharan and coworker reached an AUC of 0.74 [25]. Our XGBoost-based model achieved an AUC of 0.80 for the overall cohort as well as solely based on laboratory values and an AUC of 0.89 if no prior PCI was performed.

In terms of exact-value LVEF prediction there is no directly comparable study to our knowledge. Xin and colleagues predicted infarction size based on MRI measurements in a cohort of 315 STEMI patients without prior PCI with an impressive overall model performance (R^2^ = 0.687) [24]. Despite the fact that infarction size correlates with LVEF, there is no direct LVEF prediction, and the sample size is small, limiting the comparability with our reported results (R^2^ = 0.346 for the overall cohort). Although lactate measurements are usually quickly available in the emergency department or ICU setting, our study demonstrates that the model is capable of reliably predicting highly important clinical parameters in STEMI patients [42,43].

Several strengths of our work should be emphasized. First, to our knowledge, this is one of the largest datasets of STEMI patients analyzed with ML for LV function prediction. The size and diversity of the cohort improve the generalizability of our findings and reduce the risk of overfitting. Second, we systematically evaluated different model architectures, demonstrating the superiority of ensemble methods and confirming that even restricted feature sets (e.g., laboratory parameters only) retained predictive value. Third, we performed independent validation in a temporally distinct patient cohort from the same institution, which, despite its small size, confirmed the robustness of our findings and strengthens the translational potential of our approach.

### Limitations

Nonetheless, several limitations must be acknowledged. First, this was a single-center study, which may limit external validity given regional differences in patient populations, treatment strategies, and healthcare systems. Second, the retrospective design introduces potential bias, particularly due to the lack of standardized time points for assessing LV function and laboratory parameters. Both echocardiographic and laboratory assessments were performed during routine care, not in a protocol-driven manner, which may have introduced variability into the outcomes. Especially for lactate, a previous study has shown that lactate clearance in the first 8 h of cardiogenic shock is superior to a single admission value [42,43]. Third, although our registry initially included 2553 patients, only 1608 remained for the final analysis after applying exclusion criteria and handling missing values. This may have reduced statistical power and prevented inclusion of potentially important biomarkers such as NT-proBNP, which were not available in a sufficient number of patients [17]. Fourth, there were no standardized protocols for echocardiographic assessment of LVEF, and inter-operator variability is likely to have attenuated model performance [53,54,55].

## 5. Conclusions

ML models demonstrated that LV function at discharge and lactate dynamics after STEMI can be predicted with clinically meaningful accuracy using routinely available clinical and laboratory data. Ensemble methods, particularly Random Forest and XGBoost, consistently outperformed single algorithms and proved robust in validation. Although predictive performance was lower in patients with prior PCI, the overall findings underscore the potential of ML for individualized risk stratification after STEMI.

Importantly, an early, data-driven estimation of LVEF around the clinical cutoff of 40%, derived from routine clinical and laboratory variables, may allow rapid identification of high-risk patients even before final imaging results are available. The value of such early risk stratification is underscored by the tenfold increase in mortality associated with severely impaired LV function in the presented STEMI cohort. Our approach could thus complement echocardiography by providing an admission-based prediction of recovery potential and supporting early therapeutic and monitoring decisions.

Future research should validate these findings in larger, prospective multi-center cohorts using standardized diagnostic protocols and comprehensive biomarker assessment to establish clinical integration of ML-based prognostic tools in acute cardiac care.

## Figures and Tables

**Table 1:** Baseline characteristics. Values are shown for the full patient cohort as well as in relation to the presence or absence of a prior coronary intervention (PCI). Data are presented as mean and standard deviation (SD) or N (%) as indicated. Bold *p* values with an asterisk indicate statistical significance. BMI, body mass index; LCA, left main coronary artery; LAD, left anterior descending coronary artery; LCX, left circumflex coronary artery; RCA, right coronary artery; CAV, contrast agent volume; DAP, dose area product; CPR, cardiopulmonary resuscitation; ECMO, extracorporeal membrane oxygenation; IABP, intra-aortic balloon pump; LVEF, left ventricular ejection fraction; CK, creatine kinase; Trop, troponin; Crea, creatinine; adm, admission; max, maximum; MB, myocardial band; LDH, lactate dehydrogenase.

	Total (N = 1608)	Prior PCI (N = 325)	No Prior PCI (N = 1283)	*p* Value	Corrected *p* Value
**Demographics**					
Age [years]	64.8 (±13.5)	68.8 (±12.9)	63.8 (±13.4)	0.0000 *	0.0000 *
BMI [kg/m^2^]	27.2 (±4.4)	27.7 (±4.6)	27.1 (±4.4)	0.0123 *	0.0267 *
Sex (female) [N (%)]	404 (25.1%)	78 (24.0%)	326 (25.4%)	0.6008	0.6759
**Coronary perfusion type**					
Right [N (%)]	1401 (87.1%)	289 (88.9%)	1112 (86.7%)	0.2790	0.3720
Left [N (%)]	123 (7.7%)	20 (6.2%)	103 (8.0%)	0.2562	0.3720
Balanced [N (%)]	84 (5.2%)	16 (4.9%)	68 (5.3%)	0.7850	0.8312
**Coronary artery disease**					
1 vessel [N (%)]	497 (30.9%)	42 (12.9%)	455 (35.5%)	0.0000 *	0.0000 *
2 vessels [N (%)]	447 (27.8%)	75 (23.1%)	372 (29.0%)	0.0334 *	0.0668
3 vessels [N (%)]	664 (41.3%)	208 (64.0%)	456 (35.5%)	0.0000 *	0.0000 *
**Culprit lesion**					
LCA [N (%)]	21 (1.3%)	7 (2.2%)	14 (1.1%)	0.1317	0.2163
LAD [N (%)]	818 (50.9%)	159 (48.9%)	659 (51.4%)	0.4317	0.4317
LCX [N (%)]	176 (11.0%)	36 (11.1%)	140 (10.9%)	0.9322	0.9322
RCA [N (%)]	592 (36.8%)	122 (37.5%)	470 (36.6%)	0.7624	0.8312
**Procedural data**					
CAV [ml]	206.97 (±98.37)	188.46 (±94.29)	211.66 (±98.86)	0.0002 *	0.0006 *
Radiation time [min]	14.09 (±11.69)	14.59 (±12.98)	13.97 (±11.35)	0.5405	0.6277
DAP [cGy/cm^2^]	4306 (±4111)	4210 (±3621)	4331 (±4227)	0.3971	0.5106
Tirofiban [N (%)]	160 (10.0%)	54 (16.6%)	106 (8.3%)	0.0000 *	0.0000 *
**Hemodynamics and shock**					
Shock [N (%)]	264 (16.4%)	73 (22.5%)	191 (14.9%)	0.0010 *	0.0026 *
CPR [N (%)]	222 (13.8%)	51 (15.7%)	171 (13.3%)	0.2698	0.3720
ECMO [N (%)]	76 (4.7%)	27 (8.3%)	49 (3.8%)	0.0007 *	0.0019 *
Impella [N (%)]	15 (0.9%)	1 (0.3%)	14 (1.1%)	0.1894	0.2965
ECMO + Impella [N (%)]	4 (0.3%)	1 (0.3%)	3 (0.2%)	0.8113	0.8345
IABP [N (%)]	5 (0.3%)	0 (0.0%)	5 (0.4%)	0.2597	0.3720
LVEF discharge [%]	49 (±11)	47 (±12)	50 (±10)	0.0000 *	0.0000 *
Exitus [N (%)]	106 (6.59%)	32 (13.68%)	74 (5.39%)	0.0000 *	0.0000 *
**Laboratory values**					
CK adm [U/L]	874.7(±1501.6)	818.2 (±1685.2)	889.0 (±1451.8)	0.0001 *	0.0003 *
CK max [U/L]	2348.2 (±3726.7)	2296.4 (±4020.9)	2361.3 (±3649.9)	0.0126 *	0.0267 *
CK-MB adm [U/L]	102.1 (±162.9)	86.1 (±172.2)	106.2 (±160.3)	0.0000 *	0.0000 *
CK-MB max [U/L]	234.3 (±251.9)	206.9 (±238.6)	241.2 (±254.8)	0.0011 *	0.0026 *
Trop T adm [ng/mL]	3.41 (±10.35)	3.08 (±10.64)	3.50 (±10.27)	0.0001 *	0.0003 *
Trop T max [ng/mL]	9.09 (±15.02)	9.25 (±16.15)	9.04 (±14.73)	0.0589	0.1116
Crea adm [mg/dL]	1.13 (±0.62)	1.36 (±1.00)	1.07 (±0.46)	0.0000 *	0.0000 *
Crea max [mg/dL]	1.35 (±0.90)	1.65 (±1.32)	1.27 (±0.73)	0.0000 *	0.0000 *
LDH adm [U/L]	421.43 (±653.40)	456.82 (±760.56)	412.47 (±623.36)	0.1127	0.2029
Lactate adm [mmol/L]	2.48 (±2.47)	2.65 (±2.54)	2.44 (±2.45)	0.5278	0.6277
Lactate max [mmol/L]	3.49 (±3.80)	3.96 (±4.10)	3.37 (±3.71)	0.1322	0.2163

**Table 2:** Regression analysis for LVEF prediction. Results of the regression analysis for the prediction of the LVEF for the full cohort as well as stratified for a prior intervention and exclusively based on laboratory values as covariates. The results are compared based on mean squared error (MSE), root mean squared error (RMSE), mean absolute error (MAE), coefficient of determination (R^2^), explained variance score (EVS), and mean absolute percentage error (MAPE). The best results per subgroup are bolded. Lower values for MSE, RMSE, MAE, and MAPE indicate better performance. Vice versa, higher values for R^2^ and EVS indicate better performance. PCI, percutaneous coronary intervention; OLS, Ordinary Least Squares Regression; L1, Lasso Regularization; L2, Ridge Regularization; DT, Decision Tree; RF, Random Forest; XG, XGBoost.

		MSE	RMSE	MAE	R^2^	EVS	MAPE
**Full Cohort**	**OLS**	0.0078 (0.0064, 0.0090)	0.0881 (0.0803, 0.0950)	0.0686 (0.0619, 0.0950)	0.3202 (0.1821, 0.4354)	0.3202 (0.1915, 0.4394)	17.92% (15.89%, 20.02%)
	**OLS + L1**	0.0077 (0.0065, 0.0089)	0.0877 (0.0808, 0.0945)	0.0686 (0.0629, 0.0747)	0.3263 (0.1977, 0.4226)	0.3310 (0.2085, 0.4243)	17.08% (14.99%, 19.48%)
	**OLS + L2**	0.0076 (0.0065, 0.0090)	0.0873 (0.0809, 0.094)	0.0686 (0.0633, 0.0751)	0.3324 (0.2033, 0.4371)	0.3379 (0.2145, 0.4406)	17.02% (15.11%, 19.44%)
	**DT**	0.0095 (0.0078, 0.0113)	0.0972 (0.0882, 0.1064)	0.0754 (0.0688, 0.0826)	0.1721 (-0.0224, 0.3213)	0.1837 (0.0063, 0.3289)	17.72% (15.27%, 20.51%)
	**RF**	0.0076 (0.0063, 0.0090)	0.0873 (0.0795, 0.0950)	**0.0673** (0.0614, 0.0734)	0.3326 (0.2025, 0.4286)	0.3402 (0.2171, 0.4368)	16.11% (13.64%, 18.96%)
	**XG**	**0.0075** (0.0063, 0.0089)	**0.0864** (0.0793, 0.0942)	0.0677 (0.0620, 0.0740)	**0.3461** (0.2289, 0.4346)	**0.3542** (0.2427, 0.4414)	**16.11%** (13.89%, 18.81%)
**Prior PCI**	**OLS**	0.0144 (0.0096, 0.0173)	0.1202 (0.0980, 0.1315)	0.0953 (0.0779, 0.1057)	0.1870 (−0.1179, 0.3925)	0.1896 (−0.0894, 0.3989)	24.45% (18.24%, 30.43%)
	**OLS + L1**	0.0121 (0.0081, 0.0161)	0.1101 (0.0900, 0.1269)	0.0875 (0.0716, 0.1008)	0.2206 (−0.0787, 0.4049)	0.2213 (−0.0702, 0.4175)	26.09% (19.78%, 33.43%)
	**OLS + L2**	0.0121 (0.0081, 0.0160)	0.1098 (0.0900, 0.1265)	0.0878 (0.0719, 0.1011)	0.2097 (−0.0839, 0.3823)	0.2116 (−0.0612, 0.3988)	26.70% (20.49%, 34.19%)
	**DT**	0.0137 (0.0093, 0.0193)	0.1169 (0.0963, 0.1390)	0.0923 (0.0767, 0.1102)	0.1028 (−0.3303, 0.3722)	0.1028 (−0.2943, 0.3828)	24.43% (18.69%, 30.68%)
	**RF**	0.0122 (0.0081, 0.0165)	0.1106 (0.0901, 0.1283)	0.0882 (0.0723, 0.1043)	0.1969 (−0.1805, 0.4322)	0.1971 (−0.1578, 0.4387)	**22.81%** (17.53%, 28.60%)
	**XG**	**0.0115** (0.0080, 0.0157)	**0.1073** (0.0896, 0.1253)	**0.0850** (0.0698, 0.1015)	**0.2442** (−0.0385, 0.4156)	**0.2443** (−0.0135, 0.4229)	22.85% (16.82%, 29.78%)
**No Prior PCI**	**OLS**	0.0073 (0.0058, 0.0087)	0.0855 (0.0762, 0.0933)	0.0675 (0.0605, 0.0741)	0.3351 (0.2134, 0.4461)	0.3399 (0.2189, 0.4502)	16.27% (14.00%, 18.89%)
	**OLS + L1**	0.0073 (0.0061, 0.0085)	0.0854 (0.0781, 0.0922)	0.0679 (0.0621, 0.07327)	0.3390 (0.2203, 0.4391)	0.3435 (0.2245, 0.4408)	16.24% (14.23%, 17.42%)
	**OLS + L2**	0.0072 (0.0058, 0.0086)	0.0849 (0.0762, 0.0927)	0.0673 (0.0604, 0.0735)	0.3421 (0.2307, 0.4365)	0.3456 (0.2379, 0.4365)	16.46% (13.42%, 17.78%)
	**DT**	0.0092 (0.0074, 0.0114)	0.0961 (0.0862, 0.1068)	0.0756 (0.0684, 0.0832)	0.1766 (0.0270, 0.2993)	0.1795 (0.0286, 0.3001)	18.17% (15.67%, 20.89%)
	**RF**	**0.0072** (0.0058, 0.0086)	**0.0846** (0.0761, 0.0930)	**0.0664** (0.0604, 0.0727)	**0.3622** (0.2470, 0.4678)	**0.3627** (0.2498, 0.4707)	**15.64%** (13.61%, 17.95%)
	**XG**	0.0073 (0.0060, 0.0089)	0.0856 (0.0775, 0.0943)	0.0667 (0.0606, 0.0730)	0.3464 (0.2326, 0.4349)	0.3474 (0.2349, 0.4404)	15.79% (13.72%, 18.15%)
**Laboratory Values Only**	**OLS**	0.0088 (0.0076, 0.0101)	0.0938 (0.0873, 0.1003)	0.0760 (0.0707, 0.0819)	0.3328 (0.2322, 0.4105)	0.03345 (0.2402, 0.4139)	18.58% (16.34%, 20.98%)
	**OLS + L1**	0.0088 (0.0076, 0.0102)	0.0939 (0.0871, 0.1010)	0.0762 (0.0705, 0.0825)	0.3313 (0.2359, 0.4163)	0.3330 (0.2398, 0.4168)	18.65% (16.30%, 21.30%)
	**OLS + L2**	0.0088 (0.0076, 0.0100)	0.0938 (0.0869, 0.1001)	0.0761 (0.0706, 0.0818)	0.3328 (0.2312, 0.4135)	0.3346 (0.2387, 0.4181)	18.72% (16.21%, 21.09%)
	**DT**	0.0099 (0.0084, 0.0115)	0.0997 (0.0919, 0.1074)	0.0797 (0.0738, 0.0861)	0.2464 (0.1127, 0.3464)	0.2470 (0.1154, 0.3477)	19.68% (17.37%, 22.23%)
	**RF**	**0.0084** (0.0072, 0.0099)	**0.0918** (0.0850, 0.0994)	**0.0741** (0.0686, 0.0803)	**0.3604** (0.2564, 0.4492)	**0.3618** (0.2624, 0.4496)	**18.01%** (15.83%, 20.43%)
	**XG**	0.0085 (0.0071, 0.0097)	0.0919 (0.0844, 0.0987)	0.0738 (0.0680, 0.0797)	0.3588 (0.2559, 0.4362)	0.3594 (0.2622, 0.4387)	18.23% (15.74%, 20.51%)

**Table 3:** Classification analysis for LVEF prediction. Classification analysis for LVEF prediction for the full cohort as well as in relation to the presence or absence of a prior percutaneous coronary intervention (PCI), as well as based on laboratory values only. The results are compared based on the area under the curve (AUC), precision–recall AUC (PR-AUC), and the binary F1 score. Further, we display the binary classification results for four different LVEF thresholds (τ) in %. The best results per subgroup are bolded. LR, Logistic Regression; DT, Decision Tree; RF, Random Forest; XG, XGBoost.

		τ=30			τ=35			τ=40			τ=50		
		AUC	PR-AUC	F1	AUC	PR-AUC	F1	AUC	PR-AUC	F1	AUC	PR-AUC	F1
**Full Cohort**	**LR**	**0.53**	**0.93**	**0.95**	0.48	0.88	**0.94**	**0.82**	**0.93**	**0.90**	**0.76**	0.67	**0.62**
	**DT**	0.53	0.92	0.89	**0.50**	**0.88**	0.92	0.75	0.90	0.88	0.64	0.46	0.61
	**RF**	0.53	0.92	0.95	0.46	0.87	0.94	0.80	0.92	0.89	0.75	0.66	0.60
	**XG**	0.52	0.92	0.95	0.49	0.87	0.92	0.80	0.92	0.89	0.75	**0.67**	0.60
**Prior PCI**	**LR**	0.57	0.90	0.71	**0.68**	**0.91**	0.88	**0.78**	**0.89**	0.86	0.67	0.44	**0.50**
	**DT**	0.62	**0.92**	0.90	0.63	0.91	0.84	0.63	0.84	0.74	0.65	0.43	0.49
	**RF**	0.62	0.91	0.93	0.62	0.87	0.89	0.76	0.83	**0.88**	0.65	0.44	0.40
	**XG**	**0.66**	0.91	**0.93**	**0.65**	0.89	**0.89**	0.77	0.86	0.87	**0.69**	**0.48**	0.38
**No Prior PCI**	**LR**	0.44	0.92	0.96	0.37	0.87	0.94	0.88	**0.96**	0.91	0.75	0.69	0.65
	**DT**	0.50	**0.96**	0.93	0.52	**0.94**	0.89	0.71	0.92	0.84	0.69	0.60	0.63
	**RF**	**0.52**	0.92	**0.96**	**0.54**	0.90	**0.94**	0.84	0.94	**0.91**	**0.76**	**0.69**	**0.66**
	**XG**	0.41	0.90	0.96	0.52	0.91	0.89	**0.89**	0.95	0.89	0.75	0.68	0.61
**Laboratory** **Values Only**	**LR**	**0.77**	0.96	**0.95**	0.38	0.82	**0.93**	0.78	0.88	0.89	0.67	0.53	0.53
	**DT**	0.65	0.85	0.94	**0.51**	**089**	0.90	0.78	0.89	0.84	0.65	0.51	0.57
	**RF**	0.76	**0.97**	0.95	0.50	0.86	0.86	0.80	0.91	**0.89**	0.66	0.54	0.55
	**XG**	0.74	0.96	0.95	0.48	0.86	0.86	**0.80**	**0.91**	0.88	**0.69**	**0.55**	**0.50**

**Table 4:** Validation of LVEF prediction. Validation of the regression analysis for the prediction of LVEF for the full cohort, as well as in relation to the presence or absence of a prior PCI and based on laboratory values only as covariates. The results are compared based on mean squared error (MSE), root mean squared error (RMSE), mean absolute error (MAE), coefficient of determination (R^2^), explained variance score (EVS), and mean absolute percentage error (MAPE).

	Method	MSE	RMSE	MAE	R^2^	EVS	MAPE
**Full Cohort**	**XGBoost** **(model development)**	0.0075 (0.0063, 0.0089)	0.0864 (0.0793, 0.0942)	0.0677 (0.0620, 0.0740)	0.3461 (0.2289, 0.4346)	0.3542 (0.2427, 0.4414)	16.11% (13.89%, 18.81%)
	**Validation cohort**	0.0080	0.0894	0.0791	0.3437	0.3660	18.48%
**Prior PCI**	**XGBoost** **(model development)**	0.0115 (0.0080, 0.0157)	0.1073 (0.0896, 0.1253)	0.0850 (0.0698, 0.1015)	0.2442 (−0.0385, 0.4156)	0.2443 (−0.0135, 0.4229)	22.85% (16.82%, 29.78%)
	**Validation cohort**	0.0098	0.0988	0.0759	−0.5626	0.3594	22.55%
**No Prior PCI**	**Random Forest** **(model development)**	0.0072 (0.0058, 0.0086)	0.0846 (0.0761, 0.0930)	0.0664 (0.0604, 0.0727)	0.3622 (0.2470, 0.4678)	0.3627 (0.2498, 0.4707)	15.64% (13.61%, 17.95%)
	**Validation cohort**	0.0090	0.0948	0.0757	0.2933	0.2956	18.62%
**Laboratory** **Values Only**	**Random Forest** **(model development)**	0.0084 (0.0072, 0.0099)	0.0918 (0.0850, 0.0994)	0.0741 (0.0686, 0.0803)	0.3604 (0.2564, 0.4492)	0.3618 (0.2624, 0.4496)	18.01% (15.83%, 20.43%)
	**Validation cohort**	0.0091	0.0955	0.0852	0.2522	0.2570	20.18%

## Data Availability

Data are available on reasonable request from the corresponding author.

## Supplementary Materials

The following supporting information can be downloaded at https://www.mdpi.com/article/10.3390/jcm14238563/s1: Figure S1: Data selection flow chart.; Figure S2: Correlation matrix of predictor variables.; Figure S3: Classification performance of predicting LVEF.; Figure S4: Feature importance and SHAP beeswarm for lactate prediction.; Table S1: List of variables.; Table S2: Regression analysis of admission lactate prediction.; Table S3: Regression analysis of maximum lactate prediction.; Table S4: Categorical analysis of admission lactate prediction.; Table S5: Categorical analysis of maximum lactate prediction.; Table S6: Regression analysis of admission lactate prediction (validation cohort).; Table S7: Regression analysis of maximum lactate prediction (validation cohort).

## Abbreviations

The following abbreviations are used in this manuscript:

ACC	American College of Cardiology
AHA	American Heart Association
ALT	Alanine transaminase
AST	Aspartate transaminase
AUC	Area under the curve
BMI	Body mass index
CAD	Coronary artery disease
CAV	Contrast agent volume
CK	Creatine kinase
CK-MB	Creatine kinase-myocardial band
CMR	Cardiac magnetic resonance imaging
CPR	Cardiopulmonary resuscitation
CRP	C-reactive protein
DAP	Dose area product
DT	Decision Tree
ECG	Electrocardiogram
ECMO	Extracorporeal membrane oxygenation
EVS	Explained variance score
ICU	Intensive care unit
IABP	Intra-aortic balloon pump
ICD	Implantable cardioverter-defibrillator
LAD	Left anterior descending coronary artery
LCA	Left main coronary artery
LCX	Left circumflex coronary artery
LDH	Lactate dehydrogenase
LVEF	Left ventricular ejection fraction
LV	Left ventricle / left ventricular
MAE	Mean absolute error
MAPE	Mean absolute percentage error
MCS	Mechanical circulatory support
MI	Myocardial infarction
MINOCA	Myocardial infarction with non-obstructive coronary arteries
ML	Machine learning
MSE	Mean squared error
NT-proBNP	N-terminal pro-B-type natriuretic peptide
OLS	Ordinary Least Squares Regression
PCI	Percutaneous coronary intervention
PR-AUC	Precision–recall area under the curve
R^2^	Coefficient of determination
RCA	Right coronary artery
RF	Random Forest
RMSE	Root mean squared error
SD	Standard deviation
SHAP	Shapley additive explanations
STEMI	ST-segment elevation myocardial infarction
XG	Extreme Gradient Boosting (XGBoost)
