# Supplementary material for: Machine Learning-Based Prediction of Early Left Ventricular Function After STEMI
Source: J Clin Med. 2025 Dec 3;14(23):8563. doi: 10.3390/jcm14238563 (PMC12692794; doi:10.3390/jcm14238563)
Supplement: Supplementary file 1 [file jcm-14-08563-s001.zip › jcm-3983231-supplementary.pdf]

Supplementary Material

Supplementary Figures

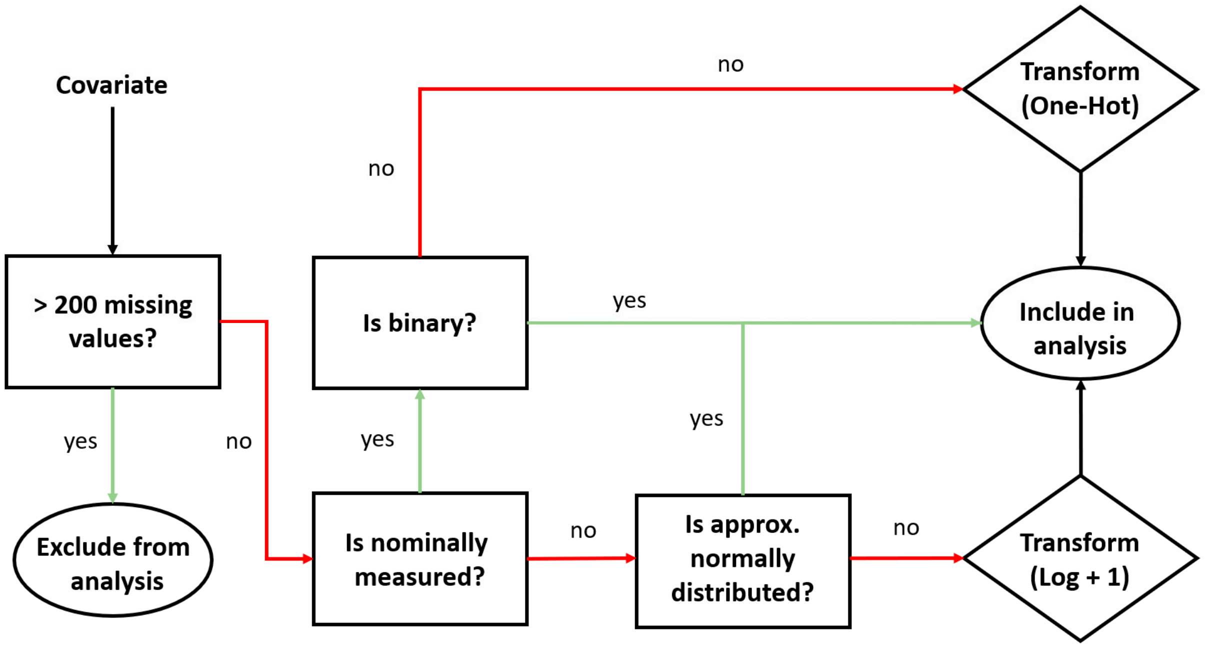

Supplementary Figure S1: Data selection flow chart.

The inclusion criterion of fewer than 200 missing values per category is illustrated, along with the downstream processing steps. Nominal covariates are one-hot encoded, whereas binary covariates are included directly. Metric covariates are log + 1 transformed when their distributions deviate substantially from normality; otherwise, they are included without transformation. Red arrows indicate that a condition is not met, green arrows denote fulfillment of the corresponding requirement (boxes), and black arrows represent the unconditional process flow.

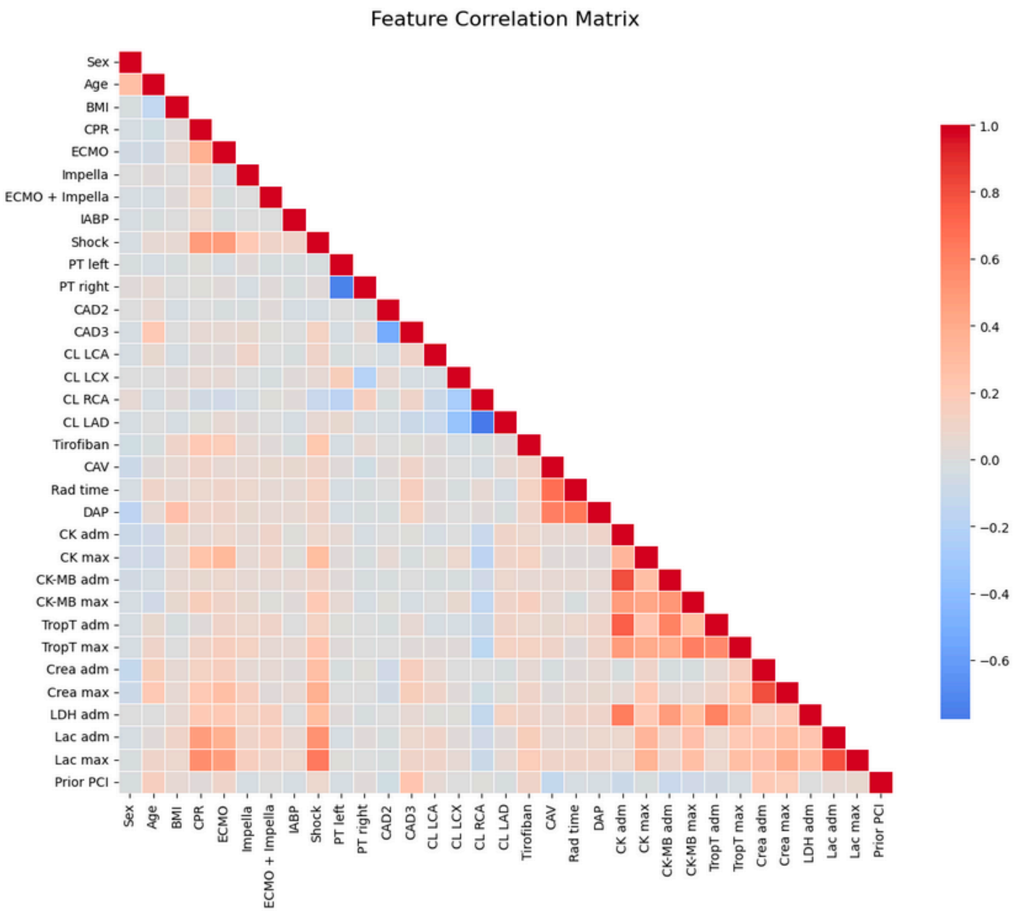

Supplementary Figure S2: Correlation matrix of predictor variables.

The figure shows pairwise associations between all predictor variables used in the analysis. Pearson correlation coefficients are reported for continuous–continuous pairs, point-biserial correlations for continuous–binary pairs, Cramér's V for categorical–categorical or categorical–binary pairs, and the phi coefficient for binary–binary pairs. BMI, body mass index; CPR, cardiopulmonary membrane oxygenation; ECMO, extracorporeal membrane oxygenation; IABP, intra-aortic balloon pump ; PT, perfusion type; CAD, coronary artery disease; CL, culprit lesion; LCA, left common coronary artery; LCX, left circumflex coronary artery; RCA, right coronary artery; LAD, left anterior descending coronary artery; CAV, contrast agent volume; DAP, dose area product; CK(-MB), creatine kinase (-myocardial band); TropT, troponin T; Crea, creatinine; adm, admission; max, maximum; LDH, lactate dehydrogenase; Lac, lactate; PCI, percutaneous coronary intervention.

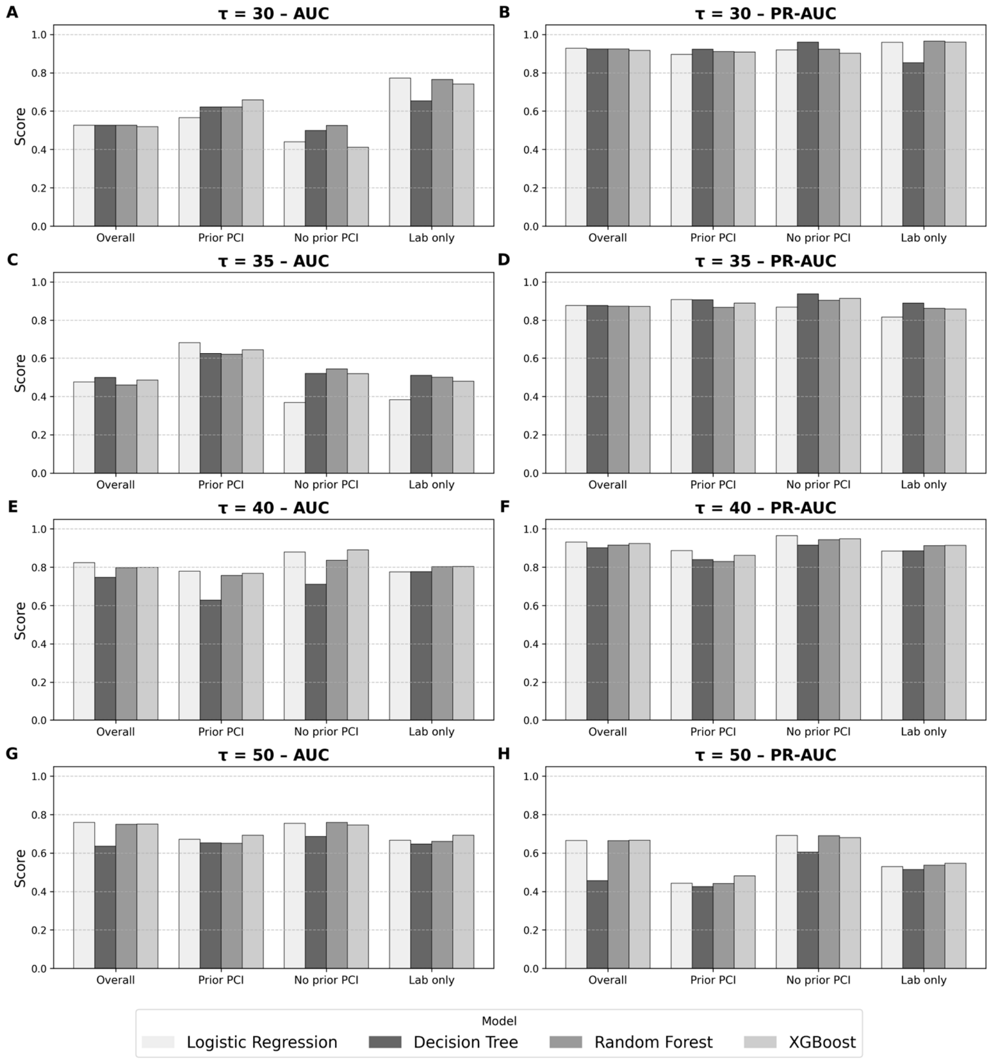

Supplementary Figure S3: Classification performance of predicting LVEF.

Classification performance with indicated models and specific left ventricular ejection fraction thresholds (τ) in %. Data is presented for the full cohort, in the presence or absence of a prior percutaneous coronary intervention (PCI) and based on laboratory values only. AUC, area under the curve; PR, precision recall.

Feature importance and SHAP visualization of log lactate predictions

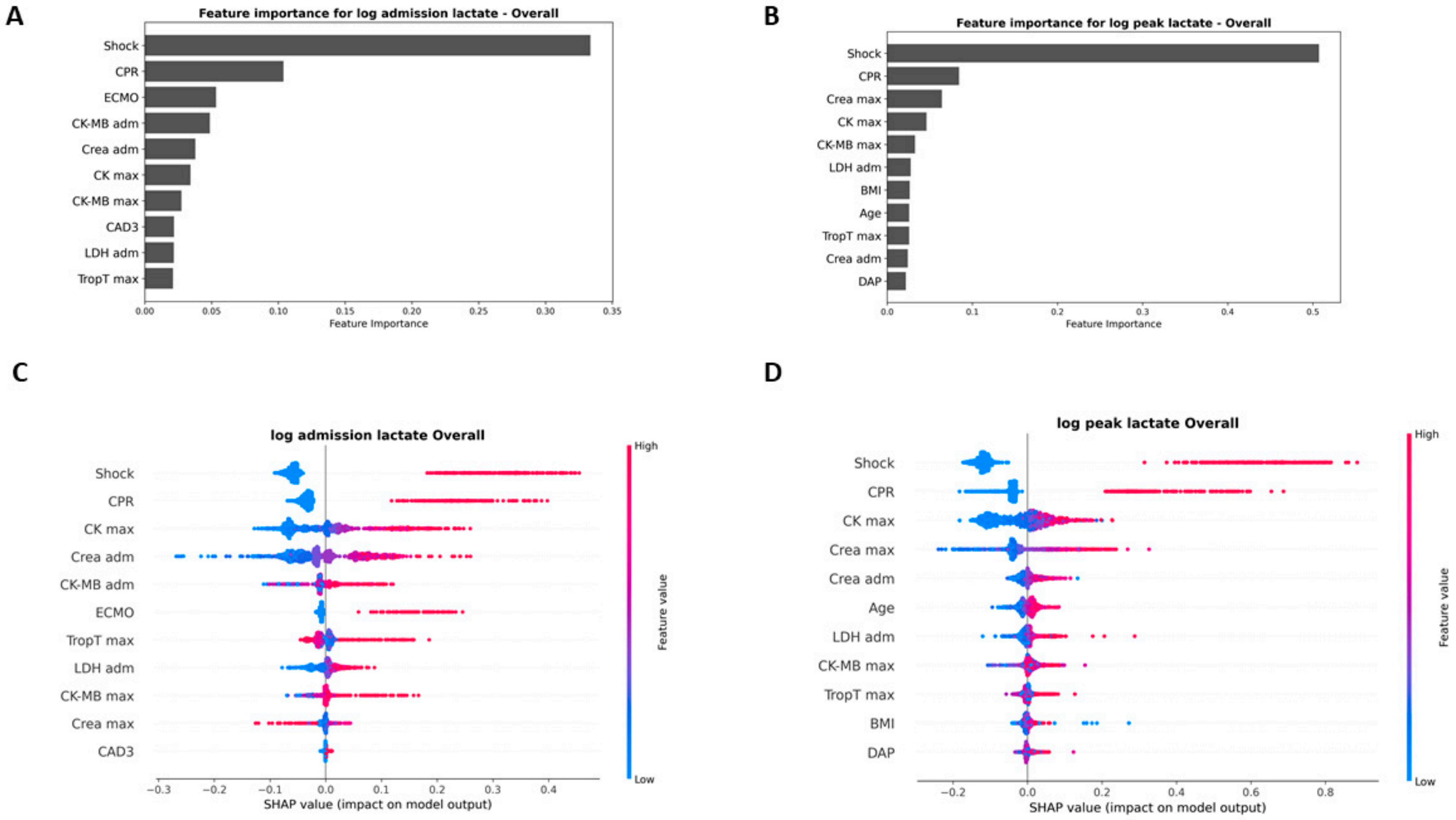

Supplementary Figure S4: Feature importance and SHAP beeswarm for lactate prediction.

Depicted are variables with highest feature importance scores with corresponding SHAP beeswam plots for (A, C) admission and (B, D) peak lactate values. All data refers to the entire study cohort. CPR, cardiopulmonary resuscitation; ECMO, extracorporeal membrane oxygenation; CK, creatine kinase; max, maximum; Crea, creatinine; adm, admission; CAD, coronary artery disease; LDH, lactate dehydrogenase; TropT, troponin T; BMI, body mass index; DAP, dose area product.

Supplementary Tables

Supplementary Table S1: List of variables.

List of all investigated variables for model development. Included variables are displayed in black, excluded variables are displayed in grey. BMI, body mass index; DAP, dose area product; CPR, cardiopulmonary membrane oxygenation; ECMO, extracorporeal membrane oxygenation; LVEF, left ventricular ejection fraction; ADP, adenosine diphosphate; LDL, low-density lipoprotein; LCA, left common coronary artery; LAD, left anterior descending coronary artery; LCX, left circumflex coronary artery; RCA, right coronary artery; IABP, intra-aortic balloon pump.

#	Variable	Category	Missing values [N (%)]	Inclusion	Level of measurement	Transformation
1	Age [years]		0	Yes	Ratio scale	Log + 1
2	BMI [kg/m^2]		0	Yes	Ratio scale	Log + 1
3	Sex (female) [N (%)]	Female	35	Yes	Nominal	One-Hot
		Male				
4	Coronary perfusion type	Left	0	Yes	Nominal	One-Hot
		Right				
		Balanced				
5	Coronary artery disease	1 Vessel	0	Yes	Nominal	One-Hot
		2 Vessels				
		3 Vessels				
6	Culprit lesion	None	0	Yes	Nominal	One-Hot
		LCA				
		LAD				
		LCX				
		RCA				
7	Contrast agent volume [ml]		11	Yes	Ratio scale	Log + 1
8	Radiation time [min]		0	Yes	Ratio scale	Log + 1
9	Dose area product [cGy/cm^2]		0	Yes	Ratio scale	Log + 1
10	Tirofiban [N (%)]	No	0	Yes	Nominal	One-Hot
		Yes				
11	Shock [N (%)]	No	0	Yes	Nominal	One-Hot
		Yes				
12	CPR [N (%)]	No	0	Yes	Nominal	One-Hot
		Yes				

13	ECMO	None<br>ECMO<br>Impella<br>ECMO+Impella<br>IABP	0	Yes	Nominal	One-Hot
14	LVEF discharge [%]		61	Yes	Ratio scale	
15	Previous coronary intervention	No<br>Yes	82	Yes	Nominal	One-Hot
16	Creatine kinase at admission		46	Yes	Ratio scale	Log + 1
17	Peak creatine kinase		53	Yes	Ratio scale	Log + 1
18	CK–MB isoenzyme at admission		180	Yes	Ratio scale	Log + 1
19	Peak creatine kinase–MB isoenzyme		93	Yes	Ratio scale	Log + 1
20	Troponin T at admission [ng/ml]		87	Yes	Ratio scale	Log + 1
21	Troponin T maximum [ng/ml]		144	Yes	Ratio scale	Log + 1
22	Creatinine at admission [mg/dl]		43	Yes	Ratio scale	Log + 1
23	Creatinine maximum [mg/dl]		40	Yes	Ratio scale	Log + 1
24	Lactate at admission		112	Yes	Ratio scale	Log + 1
25	Peak lactate		105	Yes	Ratio scale	Log + 1
26	Lactate dehydrogenase at admission		128	Yes	Ratio scale	Log + 1
27	Peak lactate dehydrogenase		272	No	Ratio scale	
28	NT-proBNP		1557	No	Ratio scale	
29	ADP		328	No	Ratio scale	
30	LDL		500	No	Ratio scale	
31	Triglycerides		486	No	Ratio scale	
32	HbA1c		663	No	Ratio scale	
33	Lipoprotein(a) nmol/l		1157	No	Ratio scale	
34	Hypertension		430	No	Nominal	
35	Hyperlipidemia		821	No	Nominal	
36	Diabetes mellitus		509	No	Nominal	
37	Family history		668	No	Nominal	
38	Nicotine intake		476	No	Nominal	

Supplementary Table S2: Regression analysis of admission lactate prediction.
Analysis of log lactate values on admission for the full cohort as well as in relation to the presence of a prior PCI (percutaneous coronary intervention). The results are compared based on mean squared error (MSE), root mean squared error (RMSE), mean absolute error (MAE), coefficient of determination (R2), explained variance score (EVS) and mean absolute percentage error (MAPE). The best results per sub-group are bolded. Lower values for MSE, RMSE, MAE and MAPE indicate better performance. Vice versa, higher values for R2 and EVS indicate better performance. DT, Decision Tree; RF, Random Forest; XG, XGBoost.

		MSE	RMSE	MAE	R^2	EVS	MAPE
**Full cohort**	**DT**	0.1635 (0.1299, 0.1993)	0.4043 (0.3604, 0.4464)	0.2987 (0.2702, 0.3270)	0.3049 (0.1698, 0.4188)	0.3070 (0.1767, 0.4221)	29.37% (26.18%, 33.32%)
	**RF**	0.1381 (0.1092, 0.1707)	0.3716 (0.3304, 0.4132)	0.2742 (0.2469, 0.3043)	0.4127 (0.3053, 0.5050)	0.4152 (0.3107, 0.5084)	**27.23%** (24.07%, 31.02%)
	**XG**	**0.1374** (0.1107, 0.1676)	**0.3707** (0.3327, 0.4094)	**0.2742** (0.2484, 0.3016)	**0.4158** (0.3178, 0.4986)	**0.4183** (0.3213, 0.5015)	27.44% (24.17%, 31.09%)
**Prior PCI**	**DT**	0.2979 (0.1766, 0.4502)	0.5458 (0.4203, 0.6710)	0.4000 (0.3174, 0.4932)	-0.4184 (-1.5552, 0.1489)	-0.3165 (-1.1704, 0.1690)	48.19% (35.32%, 62.73%)
	**RF**	0.2367 (0.1432, 0.3376)	0.4866 (0.3784, 0.5810)	0.3588 (0.2803, 0.4382)	-0.1272 (-0.8740, 0.2739)	-0.0332 (-0.5665, 0.3077)	41.35% (30.88%, 53.69%)
	**XG**	**0.2050** (0.1202, 0.3012)	**0.4528** (0.3466, 0.5488)	**0.3392** (0.2681, 0.4162)	**0.0238** (-0.5041, 0.2975)	**0.0904** (-0.2242, 0.3334)	**39.08%** (29.23%, 48.98%)
**No prior PCI**	**DT**	0.1671 (0.1337, 0.2095)	0.4087 (0.3657, 0.4577)	0.3041 (0.2724, 0.3394)	0.2625 (0.0664, 0.3887)	0.2718 (0.0718, 0.4057)	29.88% (25.95%, 34.70%)
	**RF**	0.1354 (0.1057, 0.1675)	0.3680 (0.3251, 0.4093)	0.2771 (0.2481, 0.3072)	0.4021 (0.2650, 0.5031)	0.4071 (0.2680, 0.5119)	28.01% (24.24%, 32.88%)
	**XG**	**0.1316** (0.1034, 0.1629)	**0.3628** (0.3216, 0.4036)	**0.2743** (0.2454, 0.3029)	**0.4190** (0.2682, 0.5078)	**0.4255** (0.2780, 0.5221)	**27.58%** (23.82%, 32.04%)

Supplementary Table S3: Regression analysis of maximum lactate prediction.

Analysis of maximum log lactate values for the full cohort as well as in relation to the presence of a prior percutaneous coronary intervention (PCI). The results are compared based on mean squared error (MSE), root mean squared error (RMSE), mean absolute error (MAE), coefficient of determination (R2), explained variance score (EVS) and mean absolute percentage error (MAPE). The best results per sub-group are bolded. Lower values for MSE, RMSE, MAE and MAPE indicate better performance. Vice versa, higher values for R2 and EVS indicate better performance. DT, Decision Tree; RF, Random Forest; XG, XGBoost.

		MSE	RMSE	MAE	R^2	EVS	MAPE
**Full cohort**	**DT**	0.2288 (0.1435, 0.3370)	0.4783 (0.3789, 0.5805)	0.3541 (0.2847, 0.4388)	0.3710 (0.0697, 0.5657)	0.4097 (0.1716, 0.6181)	**28.93%** (23.62%, 35.48%)
	**RF**	**0.1942** (0.1143, 0.2841)	**0.4407** (0.3381, 0.5330)	**0.3331** (0.2672, 0.4053)	**0.4660** (0.1039, 0.6815)	**0.5096** (0.2401, 0.7069)	29.57% (23.24%, 36.71%)
	**XG**	0.2085 (0.1214, 0.3088)	0.4566 (0.3485, 0.5557)	0.3477 (0.2779, 0.4239)	0.4269 (0.0699, 0.6348)	0.4629 (0.1629, 0.6716)	30.82% (24.23%, 38.43%)
**Prior PCI**	**DT**	0.2288 (0.1435, 0.3370)	0.4783 (0.3789, 0.5805)	0.3541 (0.2847, 0.4388)	0.3710 (0.0697, 0.5657)	0.4097 (0.1716, 0.6181)	**28.93%** (23.62%, 35.48%)
	**RF**	**0.1942** (0.1143, 0.2841)	**0.4407** (0.3381, 0.5330)	**0.3331** (0.2672, 0.4053)	**0.4660** (0.1039, 0.6815)	**0.5096** (0.2401, 0.7069)	29.57% (23.24%, 36.71%)
	**XG**	0.2085 (0.1214, 0.3088)	0.4566 (0.3485, 0.5557)	0.3477 (0.2779, 0.4239)	0.4269 (0.0699, 0.6348)	0.4629 (0.1629, 0.6716)	30.82% (24.23%, 38.43%)
**No prior PCI**	**DT**	0.1419 (0.0892, 0.2062)	0.3767 (0.2987, 0.4541)	0.2528 (0.2213, 0.2848)	0.5497 (0.3854, 0.6840)	0.5514 (0.3858, 0.6850)	19.45% (17.38%, 21.43%)
	**RF**	0.1432 (0.0897, 0.2127)	0.3784 (0.2995, 0.4612)	0.2554 (0.2254, 0.2912)	0.5457 (0.3745, 0.6782)	0.5458 (0.3763, 0.6787)	20.06% (18.00%, 22.15%)
	**XG**	**0.1350** (0.0815, 0.2028)	**0.3675** (0.2854, 0.4503)	**0.2459** (0.2151, 0.2786)	**0.5715** (0.4087, 0.7102)	**0.5716** (0.4105, 0.7124)	**19.41%** (17.58%, 21.41%)

Supplementary Table S4: Categorial analysis of admission lactate prediction.
Data is presented for the full cohort as well as in relation to the presence of a prior percutaneous coronary intervention (PCI). AUC, area under the curve; PR, precision recall; LR, Logistic Regression; DT, Decision Tree; RF, Random Forest; XG, XGBoost.

		$\tau = 2.5 mmol/l$			$\tau = 3.0 mmol/l$			$\tau = 3.5 mmol/l$		
		**AUC**	**PR-AUC**	**F1**	**AUC**	**PR-AUC**	**F1**	**AUC**	**PR-AUC**	**F1**
**Full cohort**	**LR**	0.5055	0.2776	0.0000	0.4672	0.1726	0.0000	**0.8784**	0.6381	0.5000
	**DT**	**0.5352**	**0.3560**	**0.2603**	**0.4864**	**0.2146**	**0.1452**	0.8182	0.6407	**0.6364**
	**RF**	0.4760	0.2715	0.0430	0.4106	0.1602	0.0000	0.8697	0.6962	0.5463
	**XG**	0.4686	0.2608	0.0000	0.4771	0.1978	0.0000	0.7639	**0.7164**	0.4928
**Prior PCI**	**LR**	0.7043	0.6840	**0.5333**	**0.5934**	**0.2800**	0.1111	0.7639	**0.5620**	**0.5263**
	**DT**	**0.7418**	**0.6169**	0.3704	0.5165	0.2672	**0.1818**	0.6744	0.3457	0.3571
	**RF**	0.6609	0.5299	0.4286	0.5742	0.2340	0.0952	**0.8277**	0.5480	0.4444
	**XG**	0.6576	0.5433	0.2500	0.5515	0.2367	0.0000	0.8009	0.5530	0.5263
**No prior PCI**	**LR**	**0.5411**	0.2756	**0.4021**	0.4822	0.1724	0.0000	0.8901	0.6706	**0.5000**
	**DT**	0.5248	**0.3415**	0.3289	0.4682	0.1690	**0.2646**	0.7825	0.5368	0.4615
	**RF**	0.5195	0.2596	0.0267	0.5197	**0.2071**	0.0000	0.8736	0.6467	0.3846
	**XG**	0.4916	0.2479	0.0000	**0.5514**	0.2036	0.0351	**0.8978**	**0.6858**	0.4528

Supplementary Table S5: Categorial analysis of maximum lactate prediction.

Data is presented for the full cohort as well as in relation to the presence of a prior percutaneous coronary intervention (PCI). AUC, area under the curve; PR, precision recall; LR, Logistic Regression; DT, Decision Tree; RF, Random Forest; XG, XGBoost.

		$\tau = 2.5mmol$			$\tau = 3.0mmol$			$\tau = 3.5mmol$		
		**AUC**	**PR-AUC**	**F1**	**AUC**	**PR-AUC**	**F1**	**AUC**	**PR-AUC**	**F1**
**Full cohort**	**LR**	0.6739	0.6593	**0.5421**	0.5138	0.3261	0.0571	**0.8419**	**0.7300**	0.6016
	**DT**	0.6489	0.6496	0.5243	**0.5467**	**0.4620**	**0.2857**	0.7401	0.6437	0.5547
	**RF**	**0.6783**	**0.6698**	0.4976	0.5320	0.3058	0.0877	0.8217	0.7160	0.6364
	**XG**	0.6729	0.6611	0.5051	0.5007	0.2977	0.2014	0.8241	0.7035	**0.6519**
**Prior PCI**	**LR**	**0.6452**	**0.6334**	0.5357	0.5161	0.4292	0.3415	0.9033	0.8622	0.6207
	**DT**	0.6442	0.6278	0.5424	0.6522	**0.5914**	**0.4651**	0.9196	0.8831	0.7619
	**RF**	0.5636	0.5431	**0.5574**	0.6107	0.4609	0.1935	**0.9348**	**0.9218**	**0.8333**
	**XG**	0.6276	0.5695	0.4906	**0.6527**	0.4670	0.2857	0.8739	0.8404	0.7368
**No prior PCI**	**LR**	0.4189	0.3387	0.0000	0.4876	0.2533	0.0000	0.8170	0.6574	**0.6383**
	**DT**	0.4259	0.3881	0.2469	**0.5559**	**0.4259**	0.3467	0.8142	0.6612	0.6167
	**RF**	**0.4877**	**0.3975**	0.2254	0.4618	0.2509	**0.6244**	**0.8363**	**0.6925**	0.5918
	**XG**	0.4417	0.3889	**0.4176**	0.4888	0.2850	0.0000	0.8223	0.6802	0.5859

Supplementary Table S6: Regression analysis of admission lactate prediction (validation cohort).

Analysis of log lactate values on admission for the full validation cohort as well as in relation to the presence of a prior percutaneous coronary intervention (PCI). The results are compared based on mean squared error (MSE), root mean squared error (RMSE), mean absolute error (MAE), coefficient of determination (R^2), explained variance score (EVS) and mean absolute percentage error (MAPE). Lower values for MSE, RMSE, MAE and MAPE indicate better performance. Vice versa, higher values for R^2 and EVS indicate better performance.

		MSE	RMSE	MAE	R^2	EVS	MAPE
**Full cohort**	**XGBoost (model development)**	0.1374 (0.1107, 0.1676)	0.3707 (0.3327, 0.4094)	0.2742 (0.2484, 0.3016)	0.4158 (0.3178, 0.4986)	0.4183 (0.3213, 0.5015)	27.44% (24.17%, 31.09%)
	**Validation cohort**	0.0800	0.2829	0.2394	0.1878	0.2683	19.62%
**Prior PCI**	**XGBoost (model development)**	0.2050 (0.1202, 0.3012)	0.4528 (0.3466, 0.5488)	0.3392 (0.2681, 0.4162)	0.0238 (-0.5041, 0.2975)	0.0904 (-0.2242, 0.3334)	39.08% (29.23%, 48.98%)
	**Validation cohort**	0.1687	0.4107	0.4029	-0.1506	-0.1145	35.95%
**No prior PCI**	**XGBoost (model development)**	0.1316 (0.1034, 0.1629)	0.3628 (0.3216, 0.4036)	0.2743 (0.2454, 0.3029)	0.4190 (0.2682, 0.5078)	0.4255 (0.2780, 0.5221)	27.58% (23.82%, 32.04%)
	**Validation cohort**	0.0490	0.2214	0.1909	0.4330	0.4926	15.65%

Supplementary Table S7: Regression analysis of maximum lactate prediction (validation cohort).

Analysis of maximum log lactate values for the full validation cohort as well as in relation to the presence of a prior percutaneous coronary intervention (PCI). The results are compared based on mean squared error (MSE), root mean squared error (RMSE), mean absolute error (MAE), coefficient of determination (R2), explained variance score (EVS) and mean absolute percentage error (MAPE). Lower values for MSE, RMSE, MAE and MAPE indicate better performance. Vice versa, higher values for R2 and EVS indicate better performance.

		MSE	RMSE	MAE	R^2	EVS	MAPE
**Full cohort**	**Random Forest (model development)**	0.1942 (0.1143, 0.2841)	0.4407 (0.3381, 0.5330)	0.3331 (0.2672, 0.4053)	0.4660 (0.1039, 0.6815)	0.5096 (0.2401, 0.7069)	29.57% (23.24%, 36.71%)
	**Validation cohort**	0.1778	0.4217	0.3123	0.4001	0.4070	21.75%
**Prior PCI**	**Random Forest (model development)**	0.1942 (0.1143, 0.2841)	0.4407 (0.3381, 0.5330)	0.3331 (0.2672, 0.4053)	0.4660 (0.1039, 0.6815)	0.5096 (0.2401, 0.7069)	29.57% (23.24%, 36.71%)
	**Validation cohort**	0.5344	0.7310	0.6373	-0.7392	-0.6212	46.34%
**No prior PCI**	**XGBoost (model development)**	0.1350 (0.0815, 0.2028)	0.3675 (0.2854, 0.4503)	0.2459 (0.2151, 0.2786)	0.5715 (0.4087, 0.7102)	0.5716 (0.4105, 0.7124)	19.41% (17.58%, 21.41%)
	**Validation cohort**	0.1161	0.3407	0.2653	0.6038	0.6070	17.72%
